# Supplementary material for: Assessment of the bacterial community structure in shallow and deep sediments of the Perdido Fold Belt region in the Gulf of Mexico
Source: PeerJ. 2018 Sep 13;6:e5583. doi: 10.7717/peerj.5583 (PMC6139248; doi:10.7717/peerj.5583)
Supplement: Supplemental Information 1 [file peerj-06-5583-s001.docx]

**Material and Methods**

**PCR and DGGE**

The extracted DNA was used as target in a nested PCR reaction to amplify the 16S rRNA gene. In the first round, the primers of the domain Bacteria 16S-F (5’-AGAGTTTGATCCTGGCTC-3’) and 16S-R (5’-CGGGAACGTATTCACCG-3’) (Ström et al., 2002) were used. The PCR was performed with an Axygen® MaxyGene™ thermal cycler (CORNING, USA) using the following conditions: an initial denaturation at 94°C/3 min, followed by 30 cycles including denaturation at 4ºC/30 sec, annealing at 52ºC/1 min and elongation at 72ºC/1 min, with a final cycle at 72ºC/10 min. The second round was conducted by a touchdown procedure described previously in Muyzer et al. (1993) using the primers 338F (5’-CCTACGGGAGGCAGCAG-3’) enriched with a 40-base GC clamp at 5’ end for further DGGE analysis, and 518R (5’-ATTACCGCGGCTGCTGG-3’), corresponding to *Escherichia coli* positions 341-534 to obtain 233 bp DNA fragments [21]. The amplification conditions were as follows: initial denaturation at 94ºC/5 min, 28 cycles including denaturation at 94ºC/1 min, annealing at 65-55ºC for 1 min (decreasing 1ºC at each cycle) and elongation 72ºC/1 min, and a final, elongation at 72ºC/10 min. All the PCR reactions (total volume reaction 25 µl) consisted of 1 x PCR buffer, 7 mM MgCl_2_, 0.2 µM dNTPs, 0.2 µM of each of the appropriate primers, 0.2 µl of Taq polymerase (Thermo Scientific) and 2 µl of total DNA at approximately 7-11 ng/µl. The re-amplifications for the second round used 1 µl of the PCR product as template. The concentration and quality of total DNA and the PCR products were checked on 1.4% agarose gels stained with ethidium bromide (EtBr).

PCR products of bacterial 16S rRNA were separated by DGGE in a DCode® Universal Mutation Detection System (Bio-Rad, USA). Similar amounts of PCR products with fragments of V3 region were applied directly onto 8% (w/v) polyacrylamide gel in a gradient of chemical denaturants between 30 to 60% (100% denaturant agent was defined as 7 M urea and 40% (v/v) deionized formamide). Electrophoresis was set at a constant condition of 50 V 60°C 18 h. The gel was stained with EtBr and archived with a UV gel documentation system (Bio-Imaging Systems, MiniBIS Pro DNNR UV, Jerusalem, Israel). Representative DGGE bands were excised with a sterile scalpel and DNA was eluted in deionized-filtered water overnight at 4ºC. DNA was re-amplified and re-analyzed by DGGE to confirm the position of the excised bands. DGGE bands were sequenced by a commercial service (GeneWiz Company, South Plainfield, USA).

**Phylogenetic analysis**

Sequence were trimmed with Chromas Lite 2.1 software http://technelysium.com.au/chromas.html(http://technelysium.com.au/chromas.htmlhttp://technelysium.com.au/chromas.html"http://technelysium.com.au/chromas.html) and compared against NCBI nucleotide database using BLAST algorithm (http://www.ncbi.nlm.nih.gov/"). DGGE band sequences and reference sequences with the highest similarity (98-100%) retrieved from the NCBI were aligned using ClustalW. A phylogenetic tree was constructed by neighbor-joining method with Jukes-Cantor substitution model using MEGA 6 package. To support the nodes, 1000 replications was performed using bootstrap analysis. Taxonomic assignation of the operational taxonomic units (OTUs) was based on BLAST and the phylogenetic analyses.

**GenBank accession numbers**

The nucleotide sequence data reported are available in the GenBank databases under the accession numbers KY323711 to KY323720.

**Functional prediction**

A functional prediction was performed with the Phylogenetic Investigation of Communities by Reconstruction of Unobserved States (PICRUST) software (Langille et al. 2013), in order to assess the metabolic pathways related to hydrocarbon and xenobiotic degradation. We filtered the OTU table to keep just the OTUs that were assigned with a GreenGenes OTU identifier by the references OTU picking step from the open references picking QIIME pipeline. The resulting OTU table was normalized with the “normalize_by_copy_number.py” script, thus the metagenome prediction was performed with the “predict_metagenomes.py” script. The resulting predicted metagenome, annotated with the Kyoto Encyclopedia Gene and Genomes (KEGG; <http://www.genome.jp/kegg/pathway/ko/>) database, was collapsed in three categories by function with the “categorize_by_function.py” script. The collapsed functions in the hierarchical classification of the KEGG database were exported to the STAMP (Parks et al. 2014) software to analyze the metabolic differences in the shallow and deep-sea samples in the Xenobiotics biodegradation and metabolism pathway category. The differences in the mean proportions between two groups was tested with a two-sided Welch’s t-test, with confidence intervals at 95%, the *p*-values were corrected with the FDR method.
