# Supplementary material for: Assessment of the bacterial community structure in shallow and deep sediments of the Perdido Fold Belt region in the Gulf of Mexico
Source: PeerJ. 2018 Sep 13;6:e5583. doi: 10.7717/peerj.5583 (PMC6139248; doi:10.7717/peerj.5583)
Supplement: Supplemental Information 2 [file peerj-06-5583-s002.html]

## This notebook have instructions to reproduce the analysis of the Peerj-23820 paper (Sánchez-Soto Jiménez *et al*., 2018)

Sometimes the supplementary files in the PeerJ platform have quotations (“) in the lines, you will need to remove this quotations in the metadata file before of replicate the analysis.

To run the code you need to install the following R package:

### phyloseq

McMurdie, P. J., & Holmes, S. (2013). phyloseq: an R package for reproducible interactive analysis and graphics of microbiome census data. PloS one, 8(4), e61217.

### vegan

Oksanen, J., Blanchet, F. G., Kindt, R., Legendre, P., Minchin, P. R., O’hara, R. B., … & Oksanen, M. J. (2013). Package ‘vegan’. Community ecology package, version, 2(9).

### DESeq2

Love, M., Anders, S., & Huber, W. (2014). Differential analysis of count data–the DESeq2 package. Genome Biol, 15(550), 10-1186.

### Importing QIIME output files to phyloseq

You need to download the supplementary files “.biom”, “.csv” and “.tree” and save it in a folder with just these files.

```
library(phyloseq)
```

```
BIOM<-merge_phyloseq(import_biom(list.files(pattern=".biom$"),list.files(pattern=".tre$")),import_qiime_sample_data(list.files(pattern=".csv")))
```

### Calculate the weigthed unifrac distance

```
WDIST<-distance(BIOM,"wunifrac")
```

```
## Warning in UniFrac(physeq, weighted = TRUE, ...): Randomly assigning root
## as -- New.ReferenceOTU37891 -- in the phylogenetic tree in the data you
## provided.
```

### Calculate the NMDS with the vegan library

```
library(vegan)
```

```
NMDS<-metaMDS(WDIST)
```

```
## Run 0 stress 0.04878132 
## Run 1 stress 0.04878153 
## ... Procrustes: rmse 0.0001884432  max resid 0.0004980447 
## ... Similar to previous best
## Run 2 stress 0.04879965 
## ... Procrustes: rmse 0.004624832  max resid 0.01175115 
## Run 3 stress 0.07020383 
## Run 4 stress 0.1174352 
## Run 5 stress 0.04879962 
## ... Procrustes: rmse 0.004633693  max resid 0.01174776 
## Run 6 stress 0.07020386 
## Run 7 stress 0.04879946 
## ... Procrustes: rmse 0.004620242  max resid 0.01177948 
## Run 8 stress 0.07020422 
## Run 9 stress 0.1415963 
## Run 10 stress 0.04878135 
## ... Procrustes: rmse 6.516389e-05  max resid 0.0001705429 
## ... Similar to previous best
## Run 11 stress 0.07020421 
## Run 12 stress 0.04878133 
## ... Procrustes: rmse 3.745313e-05  max resid 9.952078e-05 
## ... Similar to previous best
## Run 13 stress 0.04878133 
## ... Procrustes: rmse 2.639815e-05  max resid 6.894056e-05 
## ... Similar to previous best
## Run 14 stress 0.04879948 
## ... Procrustes: rmse 0.004613539  max resid 0.01180937 
## Run 15 stress 0.124432 
## Run 16 stress 0.07020385 
## Run 17 stress 0.04879949 
## ... Procrustes: rmse 0.004627016  max resid 0.01177677 
## Run 18 stress 0.1399017 
## Run 19 stress 0.1174357 
## Run 20 stress 0.1379269 
## *** Solution reached
```

### Reporting the stress value of the NMDS

```
capture.output(NMDS$stress,file="NMDS_stress_value.txt")
```

### Fitting the environmental variables with the envfit function and 10 000 permutations

```
vars_fit<-envfit(NMDS,data.frame(sample_data(BIOM)[,c("Deepth","Redox","TS","TOM","TOC","Sand","Lime","Clay")]),perm=10000)

capture.output(vars_fit,file="envfit_values.txt")
```

#### Running the PERMANOVA analysis and reporting the results

```
VARS<-data.frame(sample_data(BIOM)[,c("Deepth","Redox","TS","TOM","TOC","Sand","Lime","Clay")])

RESULTS <- lapply(colnames(VARS), function(x){
  form <- as.formula(paste("WDIST", x, sep="~")) 
  PERMAN <- adonis(form, data = VARS)
  return(as.data.frame(PERMAN$aov.tab))
}
)

capture.output(RESULTS,file="PERMANOVA_results.txt")
```

### Differental abundance analysis for OTUs by Zones

```
library("DESeq2")
```

```
DIFF <- phyloseq_to_deseq2(BIOM, ~ Zone)
```

```
## converting counts to integer mode
```

```
DIFF <- DESeq(DIFF, test="Wald", fitType="parametric")
```

```
## estimating size factors
```

```
## estimating dispersions
```

```
## gene-wise dispersion estimates
```

```
## mean-dispersion relationship
```

```
## final dispersion estimates
```

```
## fitting model and testing
```

```
RESDIFF<-results(DIFF, cooksCutoff = FALSE)
alpha<-0.01
SIG_OTUS <- RESDIFF[which(RESDIFF$padj < alpha), ]
SIG_OTUS <- cbind(as(SIG_OTUS, "data.frame"), as(tax_table(BIOM)[rownames(SIG_OTUS), ], "matrix"))

write.table(SIG_OTUS,file="Log2fold_OTUs.txt",sep="\t",quote=F)
```

You can save the below chunk of code as an R script (ej. peerj23820.R) and run it with Rscript in the same folder of the supplementary files.

```
### Usage: Rscript peerj23820.R

library(phyloseq)

BIOM<-merge_phyloseq(import_biom(list.files(pattern=".biom$"),list.files(pattern=".tre$")),import_qiime_sample_data(list.files(pattern=".csv")))

WDIST<-distance(BIOM,"wunifrac")

library(vegan)

NMDS<-metaMDS(WDIST)

capture.output(NMDS$stress,file="NMDS_stress_value.txt")

vars_fit<-envfit(NMDS,data.frame(sample_data(BIOM)[,c("Deepth","Redox","TS","TOM","TOC","Sand","Lime","Clay")]),perm=10000)

capture.output(vars_fit,file="envfit_values.txt")

VARS<-data.frame(sample_data(BIOM)[,c("Deepth","Redox","TS","TOM","TOC","Sand","Lime","Clay")])

RESULTS <- lapply(colnames(VARS), function(x){
  form <- as.formula(paste("WDIST", x, sep="~")) 
  PERMAN <- adonis(form, data = VARS)
  return(as.data.frame(PERMAN$aov.tab))
}
)

capture.output(RESULTS,file="PERMANOVA_results.txt")

library("DESeq2")

DIFF <- phyloseq_to_deseq2(BIOM, ~ Zone)
DIFF <- DESeq(DIFF, test="Wald", fitType="parametric")
RESDIFF<-results(DIFF, cooksCutoff = FALSE)
alpha<-0.01
SIG_OTUS <- RESDIFF[which(RESDIFF$padj < alpha), ]
SIG_OTUS <- cbind(as(SIG_OTUS, "data.frame"), as(tax_table(BIOM)[rownames(SIG_OTUS), ], "matrix"))

write.table(SIG_OTUS,file="Log2fold_OTUs.txt",sep="\t",quote=F)
```

#### Created by Cerqueda-Garcia D. (dacegabiol@ciencias.unam.mx)
