# Supplementary figures and images for: Assessment of the bacterial community structure in shallow and deep sediments of the Perdido Fold Belt region in the Gulf of Mexico
Source: PeerJ. 2018 Sep 13;6:e5583. doi: 10.7717/peerj.5583 (PMC6139248; doi:10.7717/peerj.5583)

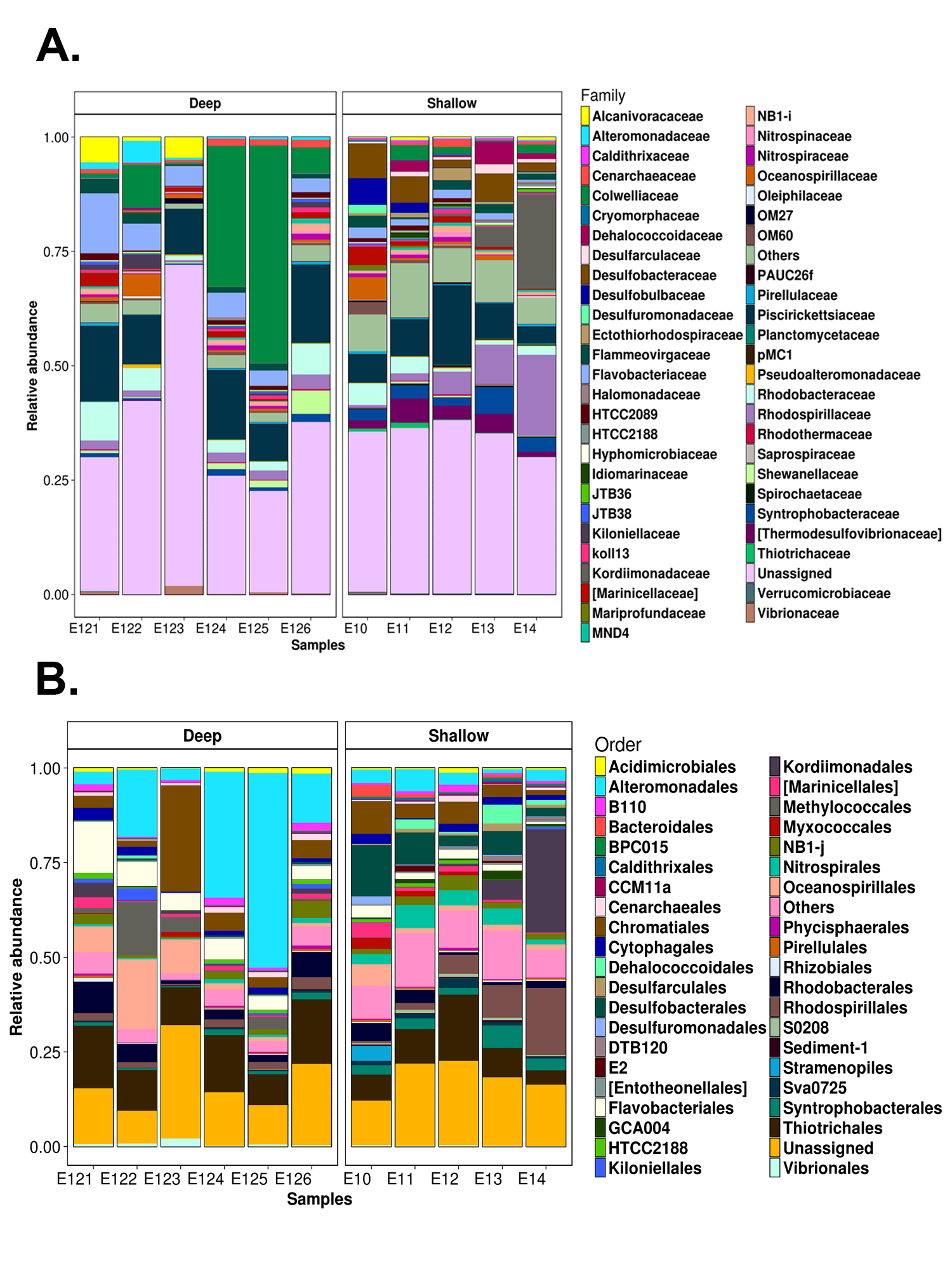

Supplement: Figure S1 [file peerj-06-5583-s003.png]

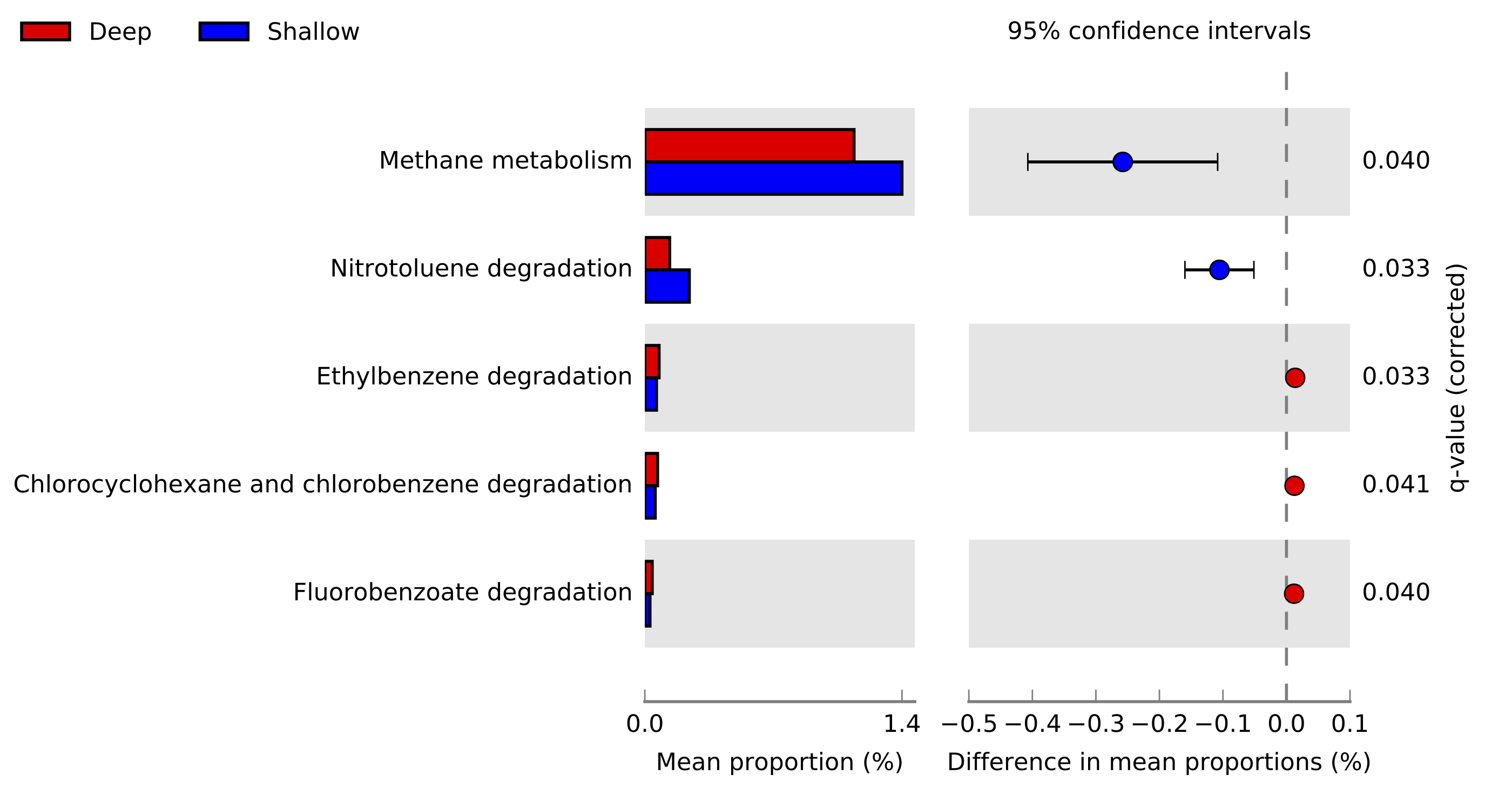

Supplement: Figure S3 — Differences in the proportions of metabolic pathways among shallow and deep-sea sediment samples were observed based on the metagenomic simulation using PICRUST software and the KEGG database. 95% confidence intervals are shown. [file peerj-06-5583-s005.png]

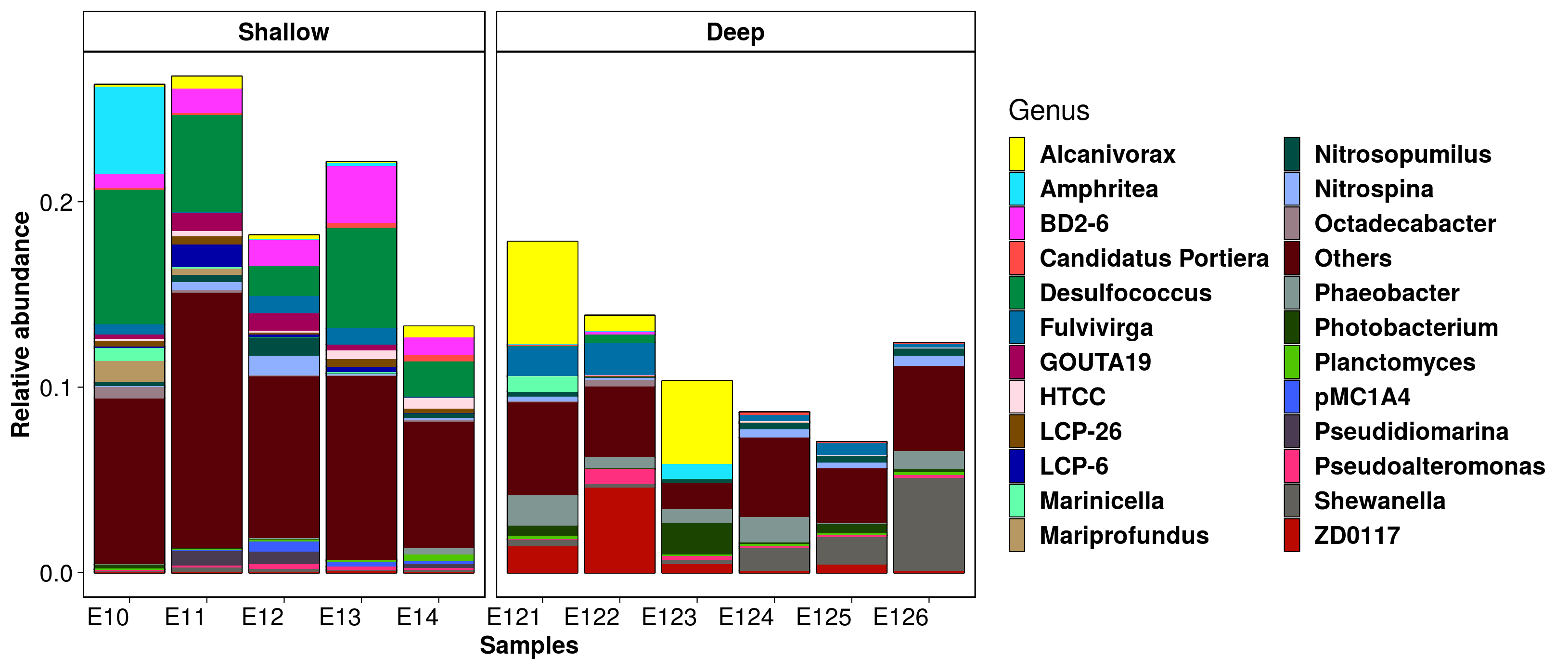

Supplement: Figure S4 [file peerj-06-5583-s006.jpg]

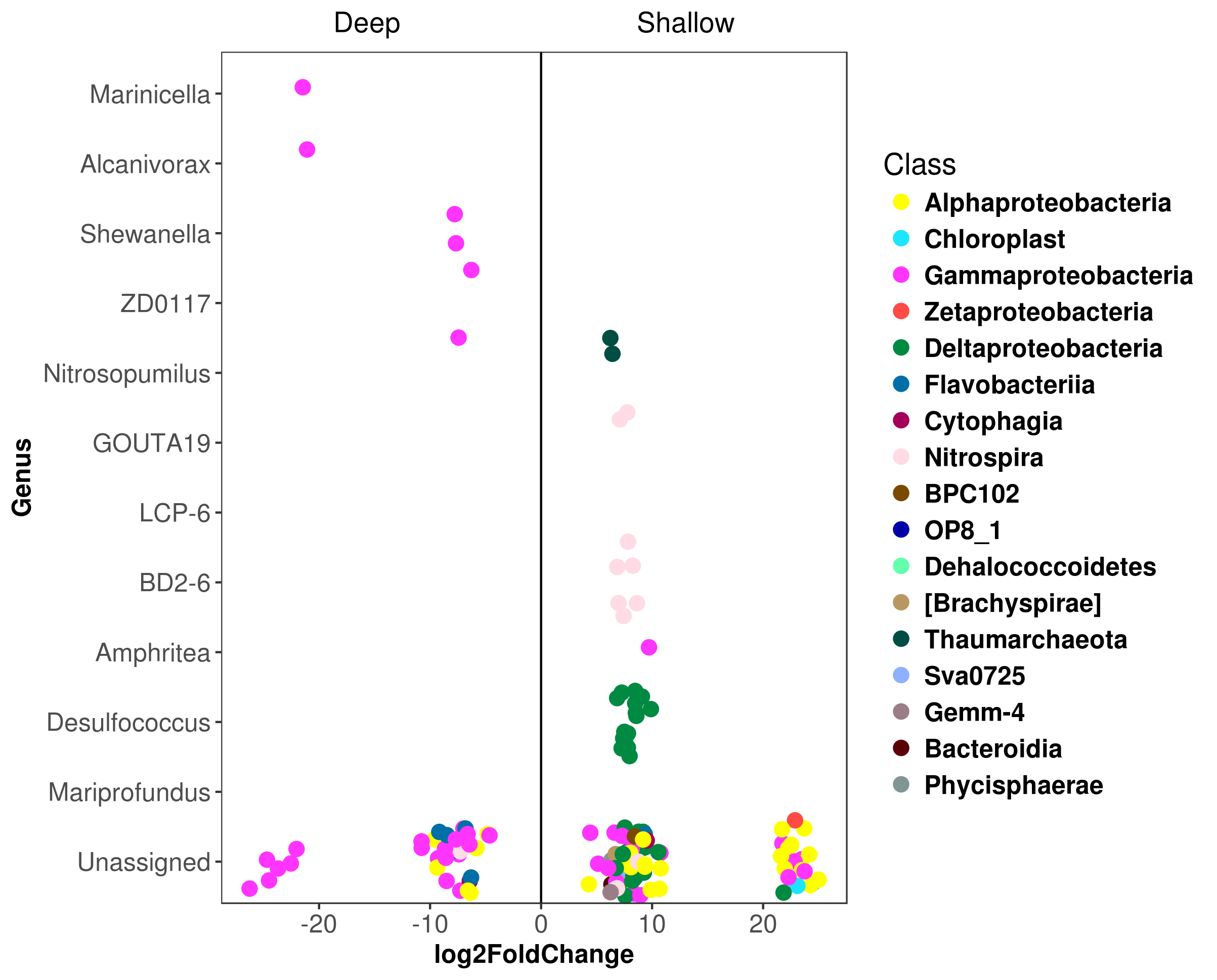

Supplement: Figure S5 [file peerj-06-5583-s007.jpg]
