# Supplementary material for: Assessment of the bacterial community structure in shallow and deep sediments of the Perdido Fold Belt region in the Gulf of Mexico
Source: PeerJ. 2018 Sep 13;6:e5583. doi: 10.7717/peerj.5583 (PMC6139248; doi:10.7717/peerj.5583)
Supplement: Table S1 [file peerj-06-5583-s008.docx]

Table S1. Permutational multivariate analysis of variance (PERMANOVA) results for the identification of physicochemical variables correlated with the microbial community structure (*p*-value <0.05).

|  |  |  |  |
| --- | --- | --- | --- |
|  | **Physicochemical variable** | **r2** |  |
|  | Depth (m) | 0.27 |  |
|  | TS (uM) | 0.27 |  |
|  | Redox potential (mV) | 0.27 |  |
|  | Clay (%) | 0.23 |  |
|  | Lime (%) | 0.2 |  |
|  |  |  |  |
