# Supplementary material for: Assessment of the bacterial community structure in shallow and deep sediments of the Perdido Fold Belt region in the Gulf of Mexico
Source: PeerJ. 2018 Sep 13;6:e5583. doi: 10.7717/peerj.5583 (PMC6139248; doi:10.7717/peerj.5583)
Supplement: Table S3 [file peerj-06-5583-s010.docx]

Table S3. Weighted nearest sequenced taxon index (NSTI) values obtained from PICRUST analysis.

| **Site** | **Sample** | **Weighted NSTI** |
| --- | --- | --- |
| **Shallow** | E10 | 0.175406364 |
|  | E11 | 0.185641263 |
|  | E12 | 0.190688132 |
|  | E13 | 0.204341884 |
|  | E14 | 0.204778131 |
| **Deep** | E121 | 0.155326798 |
|  | E122 | 0.137756874 |
|  | E123 | 0.150039232 |
|  | E124 | 0.163905173 |
|  | E125 | 0.133652615 |
|  | E126 | 0.187229322 |
