# Supplementary material for: Assessment of the bacterial community structure in shallow and deep sediments of the Perdido Fold Belt region in the Gulf of Mexico
Source: PeerJ. 2018 Sep 13;6:e5583. doi: 10.7717/peerj.5583 (PMC6139248; doi:10.7717/peerj.5583)
Supplement: Table S4 [file peerj-06-5583-s011.docx]

Table S4. Number of reads that passed >Q20 per sample. The total reads passed quality filter in this study were 457248.

| **Site** | **Sample** | **Reads (> Q20)** |
| --- | --- | --- |
| **Shallow** | E10 | 48886 |
|  | E11 | 30290 |
|  | E12 | 21243 |
|  | E13 | 35253 |
|  | E14 | 42131 |
| **Deep** | E121 | 53408 |
|  | E122 | 20402 |
|  | E123 | 61025 |
|  | E124 | 42768 |
|  | E125 | 56735 |
|  | E126 | 45107 |
