# Supplementary material for: Assessment of the bacterial community structure in shallow and deep sediments of the Perdido Fold Belt region in the Gulf of Mexico
Source: PeerJ. 2018 Sep 13;6:e5583. doi: 10.7717/peerj.5583 (PMC6139248; doi:10.7717/peerj.5583)
Supplement: Table S5 [file peerj-06-5583-s012.docx]

Table S5. Pearson correlation coefficients among environmental variables: depth (m), total sulfur (TS, uM), redox (mV), total organic matter (TOM, %), total organic carbon (TOC, uM), sand (%), lime (%) and clay (%).

| **Variable 1** | **Variable 2** | **n** | **Pearson** | ***p*-value** |
| --- | --- | --- | --- | --- |
|  |  |  |  |  |
| Depth | Redox | 11 | 0.98 | <0.0001 |
| Depth | TS | 11 | 0.98 | <0.0001 |
| Depth | TOM | 11 | -0.43 | 0.1897 |
| Depth | TOC | 11 | -0.42 | 0.1943 |
| Depth | Sand | 11 | 0.18 | 0.6041 |
| Depth | Lime | 11 | 0.78 | 0.0049 |
| Depth | Clay | 11 | -0.78 | 0.0044 |
| Redox | TS | 11 | 0.97 | <0.0001 |
| Redox | TOM | 11 | -0.38 | 0.2454 |
| Redox | TOC | 11 | -0.37 | 0.2579 |
| Redox | Sand | 11 | 0.31 | 0.3535 |
| Redox | Lime | 11 | 0.67 | 0.0232 |
| Redox | Clay | 11 | -0.76 | 0.0066 |
| TS | TOM | 11 | -0.35 | 0.2862 |
| TS | TOC | 11 | -0.35 | 0.2943 |
| TS | Sand | 11 | 0.18 | 0.6047 |
| TS | Lime | 11 | 0.74 | 0.0087 |
| TS | Clay | 11 | -0.75 | 0.0075 |
| TOM | TOC | 11 | 1 | <0.0001 |
| TOM | Sand | 11 | 0.09 | 0.7992 |
| TOM | Lime | 11 | -0.27 | 0.4274 |
| TOM | Clay | 11 | 0.19 | 0.5724 |
| TOC | Sand | 11 | 0.11 | 0.7472 |
| TOC | Lime | 11 | -0.27 | 0.4197 |
| TOC | Clay | 11 | 0.18 | 0.5894 |
| Sand | Lime | 11 | -0.07 | 0.8419 |
| Sand | Clay | 11 | -0.46 | 0.1505 |
| Lime | Clay | 11 | -0.85 | 0.0009 |
|  |  |  |  |  |
